# Cancer gene discovery in mouse and man

**DOI:** 10.1016/j.bbcan.2009.03.001

**Published:** 2009-12

**Authors:** Jenny Mattison, Louise van der Weyden, Tim Hubbard, David J. Adams

**Affiliations:** Experimental Cancer Genetics, Wellcome Trust Sanger Institute, Wellcome Trust Genome Campus, Hinxton, Cambridge, CB10 1HH, UK

**Keywords:** Cancer genome, Resequencing, Cross-species analysis, DNA copy number analysis, Mouse cancer models, Forward genetic screens

## Abstract

The elucidation of the human and mouse genome sequence and developments in high-throughput genome analysis, and in computational tools, have made it possible to profile entire cancer genomes. In parallel with these advances mouse models of cancer have evolved into a powerful tool for cancer gene discovery. Here we discuss the approaches that may be used for cancer gene identification in both human and mouse and discuss how a cross-species ‘oncogenomics’ approach to cancer gene discovery represents a powerful strategy for finding genes that drive tumourigenesis.

## 1 Genome-wide approaches for human cancer gene discovery

Tumours form when a cell gains a selective advantage over other cells and manages to evade the checkpoints that would normally suppress its growth. The acquisition of this behaviour is thought to occur as a result of the development of somatic mutations that deregulate gene function. Somatic mutations in humans may result from a multitude of genetic insults generating different types of genetic lesions. With the exception of point mutations, these lesions are rarely focal and often encompass many genes making the identification of the deregulated gene within the rearranged region problematic. In this review we will discuss the technological approaches that may be applied for finding cancer genes. An overview of these technologies is provided in Table 1.

### 1.1 Gene resequencing

Advances in DNA sequencing technology have enabled the identification of recurrent intragenic mutations across multiple cancer genomes. Davies et al. [1] screened the coding sequence and intron–exon junctions of *BRAF* for mutations in more than 900 human cancer cell lines and primary tumours, and found somatic missense mutations in 66% of malignant melanomas and in a smaller proportion of many other human cancers. 80% of *BRAF*-mutated melanomas were found to contain a V600E substitution, which is thought to constitutively activate the kinase by mimicking phosphorylation [1]. As the cost of sequencing has diminished, it has become possible to perform larger scale screens to look for mutations in multiple genes across multiple tumours. The first large-scale systematic mutational study of a complete gene family was performed by Bardelli et al. [2], who identified 7 candidate cancer genes in a screen of the tyrosine kinase gene family in 182 colorectal cancers. A further study of mutations in the tyrosine phosphatase gene family identified 6 putative tumour suppressor genes that were mutated in 26% of the colorectal cancers analysed [3]. Resequencing of the phosphatidylinositol 3-kinase (PI3K) gene family revealed one member, *PIK3CA*, which is frequently mutated in tumours of the colon, breast, brain and lung, with most mutations clustering in the catalytic domain [4]. Mutations in *PIK3CA* have since been identified in additional tumour types, such as hepatocellular carcinomas [5] and ovarian cancers [6,7]. A screen of serine/threonine kinases showed that 40% of colorectal tumours harbour a mutation in 1 of 8 PI3K-pathway genes [8]. The PI3K pathway regulates a wide range of cellular functions that are important in cancer, including growth, proliferation, survival, angiogenesis and migration [9].

Studies at the Wellcome Trust Sanger Institute have centred round the resequencing of the coding regions of all 518 protein kinase gene family members. A study of 25 breast cancers revealed diverse patterns of mutation, with a variation in the number of mutations and in the identity of mutated genes, such that no commonly point-mutated kinase gene was identified [10]. A study of 33 lung cancers reached similar conclusions [11]. While both studies showed an over-representation of nonsynonymous substitutions, which would be predicted for “driver” mutations that confer a selective growth advantage on the cancer cell, most of the mutations were thought to be “passenger” mutations that were unlikely to contribute to tumourigenesis. Protein kinase resequencing at the Sanger Institute has culminated in the identification of 921 base substitution somatic mutations in 210 diverse human cancers types [12]. Putative driver mutations were identified in 119 genes but 83% of mutations were predicted to be passengers. Cancers showed variation in mutation prevalence, with many of the cancer types with highest prevalence originating from high turnover, surface epithelia that are most exposed to mutagens [12]. Cancers also showed different “mutational signatures”, which often reflect differences in mutagenic exposure. For example, most lung cancers have a high proportion of C:G > A:T transversions, which are caused by exposure to tobacco carcinogens [11].

The first study to approach the scale of a genome-wide screen involved resequencing the coding regions of all (∼ 13,000) consensus coding sequence (CCDS) genes in 11 breast and 11 colorectal cancers [13]. Each cancer was found to harbour an average of 93 mutated genes, of which at least 11 (189 candidates in total) were thought to be driver mutations. Many of the functional groups and pathways enriched for candidate cancer genes were unique to one or other cancer type, suggesting differences in the tumourigenic process in breast and colorectal cancers [14]. There have been claims that the statistical analysis performed in this screen was flawed, in part because they used a different dataset to estimate background mutation rates, which can vary between and within cancer genomes, and because the sample size was small [15]. However, the findings of this study are in agreement with those of Greenman et al*.* [12] in suggesting that the genomic landscape of human cancers is more complex than previously thought [16]. The study has since been expanded to include all of the human RefSeq [17] genes and a larger number of breast and colorectal cancers [18]. 114 additional candidate cancer genes were identified and most candidates were mutated in fewer than 5% of tumours recapitulating the conclusions of previous studies [12,13]. Each tumour was predicted to contain an average of 15 potential driver mutations, suggesting that each mutation makes only a small contribution to tumourigenesis.

Although statistical methods can provide a prediction of the likely driver and passenger mutations within a cancer, there is a strong rationale for using functional assays to test these predictions. Frohling et al. [19] resequenced the coding exons and splice junctions of the receptor tyrosine kinase *FLT3* in samples from patients with acute myeloid leukaemia (AML). They found that out of 9 mutants with candidate driver mutations, only 4 were able to transform cells in culture (for a review, see [20]).

The Sanger Institute Catalogue of Somatic Mutations in Cancer (COSMIC) collates and displays somatic mutation information relating to human cancers [21]. At the time of writing (December 2008, COSMIC release 40), the database contained mutation data for 4773 genes from 291,551 tumours. Gene resequencing is also a major component of the $50 million 3-year pilot phase of the Cancer Genome Atlas (http://cancergenome.nih.gov/), a large-scale collaboration between the National Cancer Institute (NCI) and the National Human Genome Research Institute (NHGRI).

Gene resequencing studies have clearly been proven to be a fruitful approach for candidate cancer gene discovery. Technological breakthroughs such as ‘exon-capture’ [22], where the entire exome can be captured by array hybridization and sequenced on a parallel sequencing platform, are likely to facilitate a dramatic increase in genome-wide mutation data and will move large-scale gene resequencing studies out of genome centres and place them within reach of most research laboratories. Already this technology can be used to capture the entire human exome and, with further improvements in the reproducibility of this technology, it is likely to become the method of choice for cancer mutation screens.

### 1.2 Gene expression profiling

Gene expression arrays can be used to analyse the transcription of thousands of genes, or the entire transcriptome, simultaneously. There are two main array-based gene expression platforms: cDNA arrays, where clones corresponding to the transcripts to be analysed are spotted onto a matrix, and oligonucleotide arrays, where oligonucleotides corresponding to the transcripts are synthesised onto a matrix along with mismatch control oligonucleotides. In two-colour microarray expression analysis, the sample of interest and a control sample are differentially labelled with fluorescent dyes and are hybridized onto the array, which is then scanned to determine the ratio of fluorescence intensities for each gene. The ratio represents the relative amounts of transcript in the sample. Unsupervised clustering of the expression data for multiple samples can be used to subcategorise cancers. For example, lung cancers cluster into known histological subtypes, which are predictive of patient survival [23–25]. Gene expression profiles may also provide an indication of the genes involved in oncogenesis in a given tumour. Lung cancers harbouring a mutation in *KRAS* have a characteristic expression profile that can be used in their identification [26]. However, analysis of gene expression rarely provides insights into the underlying genetic changes and it can be confounded by physiological variation, such as the degree of inflammatory response or hypoxia [27]. Nevertheless, it is important as a complementary approach to other methods of cancer profiling, such as mutational and copy number analysis. Integrative approaches involving gene expression and copy number analysis are discussed in the following section.

Recently, a new approach for transcriptome analysis, called RNA-Seq or transcript counting, was developed. This approach involves sequencing the transcriptome of a cell or tumour on a parallel sequencing platform. Gene expression levels are calculated by ‘transcript counting’. Because many millions of reads are generated from each lane of a parallel sequencing run, RNA-Seq has a large dynamic range. Early protocols for RNA-Seq involved generating short (25–30 bp) sequence tags of the transcriptome. More recent protocols involve the use of paired-end sequencing. Paired-end sequencing greatly facilitates the generation of physical coverage of the transcriptome and, as such, can be used to identify splice variants and fusion transcripts. Because sequence data can be used for nucleotide variant calling, RNA-Seq could potentially be used to profile a tumours' pattern of mutation. The other major advantage of RNA-Seq is that it is not limited by the probes that can be tiled on an array, and as such is a potent tool for transcript discovery. The RNA-Seq approach and its application are reviewed in Wang et al. [28].

### 1.3 Analysis of DNA copy number changes

Changes in DNA copy number result from chromosomal aberrations such as deletions and duplications, non-reciprocal translocations and gene amplifications. Copy number variants (CNVs) have been identified in all humans studied [29], and a genome-wide study of 270 apparently healthy individuals from four diverse populations identified almost 1500 germline copy number variable regions encompassing 12% of the human genome [30]. CNVs have been reported to accounted for ∼ 18% of the total detected variation in gene expression between individuals, suggesting that they make a considerable contribution to phenotypic variation [31]. In the context of cancer, genomic instability results in the acquisition of somatic copy number aberrations that may contribute to tumourigenesis through the amplification of oncogenes and/or loss of tumour suppressor genes.

Chromosomal instability, which may manifest as alterations in chromosome number (aneuploidy) or inter- or intra-chromosomal rearrangement, is thought to arise early in tumourigenesis but increases with tumour progression (for a review, see [32]). There are many mechanisms that underlie this transition and cells have evolved potent checkpoints to eliminate cells with unstable genomes. Fridlyand et al. [33] found that shorter or altered telomeres were associated with a greater number of genomic amplifications and that the frequency of low-level changes was associated with altered expression of genes involved in mitosis, cell cycle, DNA replication and repair. These included many genes that are direct targets of the transcription factor E2F [33]. This has lead to the suggestion that the Rb pathway plays an important role in regulating chromosomal instability, as hypothesised by Hernando et al. [34,33]. Advanced tumours tend to reach a stable state, which, in the form of cancer cell lines, are stable over many generations and in different laboratories, suggesting that they have evolved to an optimal state [35]. It has been suggested that rather than a high level of chromosomal instability promoting tumorigenesis, highly unstable cells are selectively eliminated. This has lead to a ‘just right’ model of chromosomal instability [36].

#### 1.3.1 Using comparative genomic hybridization (CGH) to detect copy number changes

Large alterations in copy number were initially detected and quantified using metaphase spreads in a technique known as comparative genomic hybridization (CGH) [37]. In CGH, cancer and normal genomic DNA are differentially labelled with fluorochromes and are co-hybridized to normal metaphase chromosomes. Cot-1 DNA is added to suppress hybridization to repetitive elements in the genome. The ratio of fluorescence intensities at any chromosomal position is approximately proportional to the ratio of copy numbers of the cancer and normal DNA at that position (reviewed in [38]). CGH profiles can be viewed and compared using the NCBI Cancer Chromosomes database, which integrates three databases of chromosomal aberrations in cancer: the SKY/M-FISH and CGH Database, the Mitelman Database of Chromosome Aberrations in Cancer, and the Recurrent Chromosome Aberrations in Cancer Database [39]. Rearrangement breakpoints are linked to the underlying genome assembly. However, the tool is limited to cytogenetic resolution because CGH cannot detect changes of less than 20 Mb or distinguish changes that are close together, and cannot determine exact genomic coordinates [38].

Array CGH is a higher resolution, high-throughput version of conventional CGH, in which differentially labelled cancer and reference samples are hybridized to an array made from large genomic clones, e.g. bacterial artificial chromosomes (BACs), or cDNAs (for reviews, see [38,40,41]). The copy number is measured at each probe on the array, and can be mapped directly to the genome. A disadvantage of array CGH is that it cannot detect LOH, which has traditionally been identified using methods involving microsatellites and restriction fragment length polymorphisms (RFLPs) that are not suitable for large-scale analyses (see [42]).

Single nucleotide polymorphism (SNP) arrays are a more recent development in copy number analysis. SNPs, which have been reported to account for most of the genetic variation in the human genome [31], occur on average every 100–300 base pairs along the human genome sequence. The Affymetrix GeneChip Mapping Assay (http://www.affymetrix.com) is a commonly used procedure that combines a whole-genome sampling assay (WGSA) with high-density SNP arrays [43,44]. WGSA is used to reduce the complexity of the sample, and involves ligating a linker to restriction-digested DNA, which enables PCR amplification using a single primer that is complementary to the adaptor. The amplified DNA is then fragmented, labelled and hybridized to the array. SNPs within the amplified DNA are used as probes on the array, therefore maximising the amount of information that can be extracted from the experiment [45]. In the Affymetrix GeneChip Mapping 10K Assay (which uses an array containing 11,555 SNPs across the genome) WGSA involves a single restriction enzyme, XbaI [43]. Regions of the genome in which the XbaI site is rare will be under-represented in the array [45]. The higher resolution 100K SNP array therefore uses two restriction enzymes, XbaI and HindIII, which produce complementary SNP densities [44]. Each SNP in an Affymetrix array is represented by a “probe set” comprising multiple “probe quartets”. Each probe quartet consists of four 25mer oligonucleotides in the form of two “probe pairs” comprising a perfect match probe and a mismatch probe corresponding to each SNP allele. Probe quartets differ from one another in offset, i.e. the position of the polymorphic site relative to the centre of the oligonucleotide, and orientation (reviewed in [46]). Normal and tumour DNA are hybridized to different arrays, therefore avoiding the need for matched samples and allowing for a pool of normal samples to be used as a control. As in other forms of array CGH, the copy number at each probe can be inferred from the intensity of fluorescence of hybridized sample DNA [45,47].

Commercially available arrays now range in resolution from 10,000 to ∼ 1 million SNPs across the genome. SNP arrays therefore provide the potential for fine mapping of copy number changes, enabling the identification of small aberrations and accurate mapping of chromosomal breakpoints. Furthermore, the SNPs can be genotyped and compared to a normal sample to identify regions of loss of heterozygosity (LOH). This permits the identification of complex changes such as LOH without decrease in copy number and decrease in copy number without LOH [45,47,48]. Such changes are common, as demonstrated in pancreatic and cervical cancer cell lines, where the proportion of LOH associated with copy-reduction was found to be just 32% [49] and 25% [50], respectively. Allele-specific amplification can also be detected using SNP arrays.

CGH signal intensities must be normalised to account for technical bias while still retaining biologically relevant changes. Normalisation of array CGH data has generally involved the use of methods originally developed for normalising gene expression microarray data (for a review, see [51]). Cross-slide and within-slide normalisation are used to transform the data such that all arrays, and all the spots on each array, are comparable. In median normalisation, all values are multiplied by a constant factor so that all arrays have a median log_2_ ratio of 0. Lowess, or Loess, normalisation accounts for spot intensity biases and other dependencies such as the location of the spot on the array and the use of different print tips. The data are linearised by subtracting a Lowess regression curve. A number of additional methods for dealing with spatial effects in expression microarray data are reviewed in Neuvial et al. [52].

In general, array CGH must be more stringent than gene expression analysis because it is required to detect single copy changes and, while the copy number of a gene, unlike the expression level, is expected to be identical in two samples, this is often not the case due to tumour heterogeneity and the presence of contaminating stromal cells [53]. Khojasteh et al. [53] proposed a multi-step normalisation process specifically for dealing with array CGH data. A “spatial segmentation” algorithm has also been developed to account for array CGH-specific spatial effects designated “local spatial biases”, where clusters of spots show a shift in signal, and “continuous spatial gradient”, where there is a smooth gradient in signal across the array [52]. Staaf et al. [54] showed that copy number imbalances correlate with intensity in array CGH data and that normalisation of expression data erroneously corrects for biologically relevant gains in copy number. They have therefore developed a normalisation algorithm that prevents suppression of copy number ratios by stratifying the data into separate populations representing discrete copy number levels [54]. Array CGH data are also affected by a genome-wide technical artefact termed “spatial autocorrelation”, or “wave”, for which the peaks and troughs are aligned across samples but the amplitude, and for some samples, the direction, varies [55]. Removal of the wave using a Lowess curve led to an increase in the number of biologically relevant CNVs detected in array CGH data from normal individuals [55].

Affymetrix has developed a number of procedures for normalising SNP array CGH data. As described above, each SNP on an Affymetrix array is represented by a probe set comprising multiple probe pairs. Fluorescence on the mismatch probes represents non-specific hybridization, and the data can be corrected by subtracting the mismatch from the perfect match intensity for each probe pair. The corrected intensities are then averaged across the probe set. The data can be globally normalised by multiplying the average intensity of the experimental array, i.e. the array to which the cancer sample is hybridized, by a normalisation factor to make it numerically equivalent to the average intensity of the control array, to which a normal sample is hybridized. Intensity ratios are calculated by dividing the average intensity for each SNP in the experimental array by the equivalent value in the control array. Three software packages that are commonly used for processing copy number data on Affymetrix SNP arrays are Copy Number Analyser for GeneChip arrays (CNAG) [CNAG, 56], DNA-Chip Analyzer (dChip) [dChip, 47] and Affymetrix GeneChip Chromosome Copy Number Analysis Tool (CNAT) (CNAT, [57]). These are compared and reviewed in Baross et al. [58], who concluded that the detection of all real CNVs from a 100K array necessitated the combined use of multiple procedures. The CNAG tool corrects for inter-experimental variation in PCR kinetics by compensating for differences in the length of PCR fragments and GC content, and uses the average of at least 5 “best-fit” normal samples that show the least variation between arrays as a control [56]. A recently published algorithm, ITALICS, addresses the problems of PCR kinetics plus additional sources of nonrelevant variation between the probe quartet intensities, such as systematic variation and spatial effects, and uses an iterative normalisation approach to estimate and remove the nonrelevant effects from the estimated biological signal [59].

ITALICS uses the GLAD algorithm [60] to estimate the biological signal by inferring the copy number across the genome [59]. GLAD is one of many methods that have been developed for segmenting the genome into regions of homogeneous copy number. Different approaches have been used, including change-point analysis, where the genome is segmented at points in the genome where the copy number changes significantly [61,62], Hidden Markov Models (HMMs) [56,63–67], hierarchical clustering along chromosomes [68] and smoothing methods [69,70]. There are also a number of web-based applications, such as ADaCGH [71] and CGHweb [72], for viewing and comparing outputs from multiple algorithms. Further methods have been developed to identify copy number changes specifically in SNP array CGH data, which has increased noise at the probe level compared with BAC array CGH [73], and a number of these infer allele-specific copy numbers [56,73–76].

Finally, having identified regions of copy number change, the statistical power can be increased by examining the region across many samples. Unlike for CNVs in normal samples, cross-sample analysis of copy number changes in cancer is hampered by the large size of many rearrangements, variation in the location of breakpoints between samples, and sample heterogeneity that prevents accurate estimation of the copy number [55]. A handful of methods have been developed to identify recurrent regions of copy number change in tumours: CMAR [77], STAC [78], H-HMM [79] and KC-SMART [80]. The latter is the only algorithm that does not discretise the data into 3 states (1, 0, − 1), which can lead to undetected copy number changes in heterogeneous tumours, and it enables the analysis of both large and small aberrations [80].

#### 1.3.2 Analysis of copy number changes in cancer genomes

CGH can detect aneuploidy, gene amplifications and deletions, and non-reciprocal translocations in cancer genomes. Gene amplifications are gains in copy number of restricted regions of DNA [81] that contribute to tumourigenesis by increasing the transcript levels, and therefore the protein levels, of oncogenes [82]. Gene amplification is the major mechanism of oncogenesis for a number of cancer genes, including *MYCN*, which is amplified in ∼ 30% of advanced neuroblastomas [83]. Amplified genes represent a promising target for cancer therapy, as demonstrated in breast cancers harbouring an amplified *HER*/*ERBB2* receptor [84].

Deletions are an important mechanism for inactivating tumour suppressor genes, including *PTEN* [85] and *CDKN2A* (*INK4A/ARF*) [86]. A genome-wide analysis of homozygous deletions in over 600 cancer cell lines showed that deletions occur in regions with fewer genes and repeat elements but higher flexibility compared with the rest of the genome [87]. A significant proportion occur in regions that are prone to chromosome breakage, and some of the genes in these “fragile sites”, such as *WWOX* and *FHIT*, show similar mutational patterns to known tumour suppressor genes, so it is not clear whether or not these genes are causally implicated in cancer [88].

Like gene expression analysis, copy number profiling can be used to subcategorise cancers. It can distinguish three subtypes of glioblastoma [89], and separates leiomyosarcomas into a distinct cluster from gastrointestinal stromal tumours, which, until recently, were classified as the same tumour type [90]. It also provides predictive power in breast cancer prognosis, where a poor prognosis is indicated by high-level amplification [91], extensive chromosome instability [33] and/or the presence of multiple, closely spaced amplicons, or “firestorms”, on a single chromosome arm [92]. Copy number profiles can also help to stage a tumour, such as in cervical cancer, where gain of chromosome 3q is associated with the transition from severe dysplasia to invasive carcinoma [93]. Furthermore, studies in ovarian cancer have revealed an association between drug response and the presence of copy number changes associated with drug sensitivity or resistance [94,95]. The amplification of genes involved in drug metabolism or inactivation is commonly observed in cultured cells as a means of acquiring drug resistance [32].

While many cancer genomes have been analysed for copy number changes, there has been limited progress in identifying the functional significance of altered regions. One successful approach involves identifying recurrently altered regions that are specific to particular tumour types. This enables the identification of “lineage addiction” cancer genes, which may target essential lineage-specific survival functions and therefore represent promising therapeutic targets [96]. Two such genes are the melanoma-specific oncogene *MITF*, which is selectively amplified and overexpressed in 20% of melanomas [97], and *NKX2-1*, which lies in the minimal amplified region of a lung-cancer-specific 14q13.3 amplicon found in up to 20% of lung cancers [98,99]. Genes *TTF1* and *NKX2-8* are usually co-amplified with *NKX2-1* in the 14q13.3 amplicon and all three genes have been shown to co-operate in lung tumourigenesis [99]. The co-occurrence and mutual exclusivity of copy number alterations at different loci may also reflect co-operating and complementary cancer genes, respectively. For example, gains of *ERBB2* and *CCNE1* frequently co-occur in bladder cancer, while *CCND1* and *E2F1*, which function in the same pathway, never co-occur [100].

The identification of cancer genes in regions of copy number change can be challenging because changes often span large regions of the genome that encompass many genes and may include many attractive candidates. Gains of more than one copy may have involved multiple evolutionary events and the critical gene may reside at the highest peak in copy number, as demonstrated for oncogenes *CYP24* and *ZNF217* in breast cancer [101]. Measurement of gene expression is also important for evaluating candidate cancer genes. *SPANXB* was identified as the putative critical gene in an Xq duplication in acute lymphoblastic leukaemias with an *ETV6/RUNX1* translocation as a result of high and uniform overexpression across all samples [102]. While gene expression and gene dosage are rarely perfectly correlated, many studies, such as the comparison of array CGH and gene expression data in breast cancers, have shown good correlation [103,104]. However, genes that are amplified are not necessarily overexpressed, as demonstrated by Kloth et al. [50], who did not observe a genome-wide correlation between copy number and gene expression in cervical cancer cell lines. Gene expression is influenced by factors other than gene dosage, such as the availability of transcription and regulatory factors, DNA methylation and chromatin conformation, and the presence of miRNAs [50].

The integration of copy number analysis with gene resequencing also facilitates cancer gene identification. Mullighan et al. [105] performed a genome-wide analysis of genetic alterations in 242 paediatric acute lymphoblastic leukaemias (ALL) using 100K and 250K SNP arrays. They found mutations in genes that regulate late B lymphocyte development in 40% of B-progenitor ALL cases. *PAX5* mutations, which included deletions, point mutations and translocations, were identified in 32% of cases [105]. ALL genomes are relatively stable, but genomes harbouring different translocations show variability in the number of copy number changes, which may reflect differences in the number of events required for tumourigenesis [105,106]. The integration of resequencing data, and epigenetic data, can facilitate the identification of tumour suppressor genes in regions of LOH, where the other allele may be inactivated by point mutation or epigenetic changes.

The identification of human cancer genes is aided by the integration of a number of complementary genome-wide analyses of human cancers, but the integration of cancer-associated mutation datasets from other species, particularly the mouse, provides an even more powerful approach for cancer gene discovery. Cross-species cancer gene analysis is discussed in Section 3.

#### 1.3.3 Limitations of CGH and alternative strategies

Limitations of CGH-based approaches include the inability to determine the ploidy of the sample or to identify the location of rearranged sequences in the cancer genome. However, the ploidy and location of larger rearrangements (> 10 Mb) can be discerned by combining CGH with G-banding or Spectral Karyotyping (SKY) [107]. CGH may also struggle to detect low-level changes and changes in heterogeneous samples, e.g. primary cancers containing normal stromal cells, and it is affected by low-copy reiterated sequences, including gene paralogues (for a full review, see [108]).

A further limitation of CGH is that while it can detect non-reciprocal, or unbalanced, translocations, which result in the gain or loss of DNA and often cause the inactivation of tumour suppressor genes [109], it cannot detect reciprocal, or balanced, translocations. These result in fusion transcripts or transcriptional deregulation due to the positioning of an intact gene next to the promoter or enhancer elements of another gene. Until recently, it was thought that balanced translocations predominated in haematopoietic tumours, but an assessment of data in the Mitelman Database of Chromosome Aberrations in Cancer suggests that they also play an important role in epithelial tumourigenesis [109]. Furthermore, human solid tumours appear to contain large numbers of gene fusions [110] and a quarter of the breakpoints detected in 3 breast cancer cell lines were found to be balanced [111].

Balanced translocations are often initiating events in tumourigenesis that are essential for tumour development, and they therefore represent promising therapeutic targets. However, the high-throughput identification of balanced translocations has been hindered because translocation breakpoints cannot be amplified by PCR [111]. Genome-wide techniques for identifying translocations include array painting, in which chromosomes are sorted and DNA is amplified and hybridized to DNA microarrays [111], and informatics approaches, such as the algorithm developed by Tomlins et al. [112] that used RNA expression data to identify candidate gene fusions in prostate cancers. The *EML4*-*ALK* fusion was identified in non-small-cell lung cancers by paired-end sequencing [113].

**Fig. 1:**
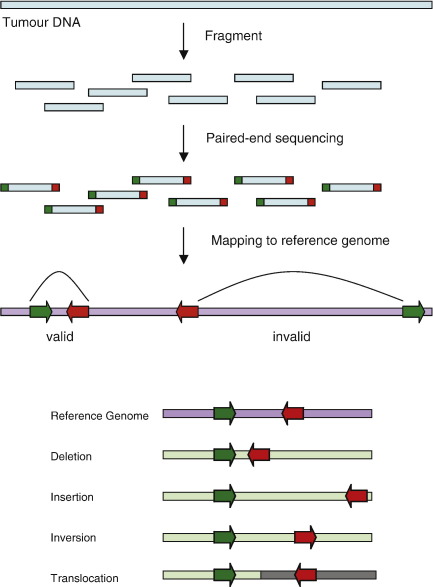
End-sequence profiling of tumour DNA. The tumour genome is fragmented and the ends of the fragmented DNA molecules are sequenced. These sequenced ends are then mapped to the reference genome. Ends that are an abnormal distance apart, or in an abnormal orientation, shown here as “invalid”, are indicative of rearrangements within the tumour genome. Redrawn with modifications from Raphael et al. [117].

End-sequence profiling (ESP) can be used to precisely map all types of genomic rearrangements, including balanced translocations [114] (Fig. 1). ESP involves constructing a BAC library from the cancer genome and sequencing the ends of clones to identify rearrangements, which map to locations in the reference genome that are of abnormal distance or orientation [114]. DNA may also be sheared and end-sequenced on a parallel sequencing platform [115]. The method can also identify fusion transcripts (tESP) and can be targeted to specific amplicons [110]. Complete sequencing of the BACs enables detailed analysis of the structure of genomic rearrangements, enabling elucidation of the mechanisms of rearrangement. ESP-based analysis of 4 cancer amplicons revealed evidence for sister chromatid break–fusion–bridge cycles, the excision and reintegration of double minutes (extrachromosomal DNA), and more complex architectures involving clusters of small genomic fragments [81]. Break–fusion–bridge cycles are initiated by a double-strand chromosomal break, which, following DNA synthesis, results in sister chromatids with identical free DNA ends that fuse to one another to prevent apoptosis. An anaphase bridge is formed during chromatid separation in mitosis, and this results in a new double-strand break and reinitiation of the cycle [116].

ESP analysis of 6 epithelial cancers, including primary tumours from brain, breast and ovary, plus a metastatic prostate tumour and 2 breast cancer cell lines, revealed extensive chromosomal rearrangements, some of which appeared to be recurrent [117]. However, ESP is not suitable for analysing large numbers of cancer genomes. A high-throughput approach, which involves massively parallel sequencing of the ends of randomly sheared DNA, has recently been applied to the genome-wide analysis of somatic and germline rearrangements in 2 lung cancers [115]. The analysis revealed a wide spectrum of rearrangements, as well as providing high-resolution copy number information. Despite the benefits of this strategy, sequencing large numbers of clones across many cancer genomes is costly and impractical. However, Bashir et al. [118] have derived a formula to maximise the probability of detecting fusion genes with the least amount of sequencing. The formula depends on the distribution of gene lengths and the parameters of the sequencing strategy used [118]. Paired-end sequencing is an attractive strategy for the complete characterisation of rearrangements in cancer. However, the recent discovery that cytogenetically balanced translocations are frequently associated with focal copy number alterations suggests that high-resolution array CGH may in fact be suitable for detecting most translocations in cancer [107].

### 1.4 Sequencing whole cancer genomes

With the advent of new sequencing technologies it is now possible to screen the cancer genome for rearrangements [115] and to sequence entire genomes [119,120]. This technology has rapidly been applied to the study of the cancer genome [121]. The first cancer genome to be sequenced was that of an acute myeloid leukaemia with a normal karyotype [121]. This study identified mutations in a number of previously known cancer genes and several mutations in novel genes, which were investigated in a larger set of acute myeloid leukaemias finding that many of these mutations were unique to the sequenced cancer. The short read technology used in these human genome sequencing projects is ideal for finding structural rearrangements and for nucleotide variant calling in the non-repetitive regions of the genome, but resolving entire genomes to the quality of the human genome assembly is not possible with this technology due to the repetitive nature of the human genome and the significant level of rearrangement normally found in human cancers. To completely sequence an entire cancer genome, application of some of the new experimental long read sequencing technologies will need to be achieved. The other confounding factor in the analysis of tumours is that they exhibit significant molecular heterogeneity adding to the complexity of deciphering the cancer genome and making cancer genome sequencing a significantly more complex undertaking than sequencing genomes to assess germline variation. Despite these difficulties, sequencing whole cancer genomes is clearly a major advance from which we will learn a significant amount about the genes involved in cancer formation and how the cancer genome evolves. Recently, the International Cancer Genome Consortium was formed to co-ordinate the sequencing of human cancer genomes. This ambitious project will sequence the genomes of all of the major cancer types and will undoubtedly precipitate a revolution in how we think about cancer and the genes that drive its development.

### 1.5 Epigenetic profiling

Epigenetic changes are chemical modifications to the DNA or histones that change the structure of chromatin but do not alter the DNA sequence. If chromatin is in the condensed conformation, transcription factors cannot access the DNA and genes are therefore not expressed, whereas genes in open chromatin can be expressed as required. DNA methylation and changes in chromatin conformation have both been implicated in tumourigenesis. DNA methylation of CpG islands, which are located in promoter regions, can result in gene “silencing” by preventing transcription factor binding. It can also repress gene expression by recruiting methyl-binding domain proteins, which associate with histone deacetylases (HDACs). HDACs mediate chromatin condensation by deacetylating histones. [122].

Aberrant DNA methylation of *CDKN2A* has been observed in a wide range of common cancer types [123,124], while *VHL* and *BRCA1* are silenced by methylation in a significant proportion of kidney [125] and breast and ovarian cancers [126], respectively. *VHL* and *BRCA1* are also frequently mutated in cancer, but for other tumour suppressor genes, such as *RASSF1A*, promoter hypermethylation appears to be the principal mechanism for inactivation (for a review, see [127]).

Methods involving high-density oligonucleotide arrays have been developed for genome-wide detection of epigenetic changes. Detection of DNA methylation relies on the ability to distinguish cytosine from 5-methylcytosine, while histone modifications can be detected using chromatin immunopreciptation (ChIP). Large genomic regions, such as an entire chromsome arm, can show aberrant methylation in cancer [128], and there is evidence to suggest that some cancers show a CpG island methylator phenotype (CIMP). CIMP + colorectal cancers have significantly more hypermethylation at CpG islands, including an increased incidence of *CDKN2A* and *THBS1* methylation [129], and they are characterised by a methylated mismatch repair gene, *MLH1*, which gives rise to microsatellite instability [130]. Genes that are reversibly repressed by Polycomb proteins in embryonic stem cells are significantly over-represented amongst constitutively hypermethylated genes in colorectal cancers [131]. This provides support for the theory of a stem cell origin of cancer. A detailed discussion of the epigenomics of cancer is beyond the scope of this review, which focuses on changes in cancer that alter the DNA sequence. Epigenomics approaches are reviewed in Callinan and Feinberg [132] and, for a detailed review of epigenomics and its relevance to the cancer stem cell hypothesis, see Jones and Baylin [133].

### 1.6 Insertional mutagenesis and cancer gene discovery in human

In the proceeding section of this review we will discuss the application of mouse models for cancer gene discovery, including how insertional mutagenesis has been deployed to find cancer genes. It is important, however, to note that insertional mutagenesis is not confined to experimental organisms, and is a mechanism of cancer initiation in humans.

There are several oncogenic viruses that afflict humans including the human papilloma virus, the Human T-cell Lymphotropic Virus (HTLV1), the hepatitis family of viruses, and the human immunodeficiency virus (HIV) that have all been implicated as insertional mutagens. In each case, it has been shown that the virus may integrate near cancer related genes, although whether clonal insertion events occur is unclear [134–137]. Insertional mutagenesis in humans has been proven to occur in patients who have received retroviral therapy for SCID-X1. Some of these patients developed T-ALL after having received an autologous transplant of cells transduced with a retrovirus expressing a wildtype copy of the γc gene, which is mutated in SCID-X1. Several of these patients acquired clonal viral insertions upstream of *LMO2*, implicating this gene as an oncogene [136]. Recently it was suggested that transcriptional upregulation of *LMO2* by retroviral insertions alone was insufficient for cancer to form, and that alterations in other genes such as *NOTCH1* were required [138].

### 1.7 Using pathways to predict cancer genes and their function

In this review, we have largely focused on gene discovery by looking for mutated, silenced, or rearranged genes. An alternative way of discovering cancer genes is to build pathways around them and to examine how they are ‘connected’ to each other and how they participate in a biological process. This approach is called ‘network modelling’. By combining gene expression data, functional genomic data and proteomic data, components of the network can be linked. This approach was shown to link breast cancer susceptibility to centrosome dysfunction in tumours carrying *BRCA1* mutations, and importantly identified the *HMMR* gene as a new breast cancer susceptibility gene [139]. An alternative approach is to develop ‘module maps’ which cluster genes together based on their behaviour or expression. Recently it was shown that genes may be clustered together to form an embryonic stem cell module map, and that expression of genes that form this signature of ‘stemness’ is predictive of decreased survival in both mouse and human cancers [140,141]. Similarly, genes may be implicated as possible cancer genes based on their functional or physical interaction with known cancer genes [142].

## 2 Cancer gene discovery in the mouse

### 2.1 The mouse as a model for studying cancer

The mouse is a leading model system for cancer research because it has a rapid reproduction rate, breeds well in captivity, and, owing to its small size, can be maintained in large numbers in limited space. It is also genetically and physiologically similar to humans. Additionally, the mouse genome has been sequenced and annotated to a high standard, second only to that of human (see [143]).

The mouse was initially used for tumour transplantation within inbred strains, but following the discovery of the immunodeficient “nude” mouse and, later, the severe combined immunodeficient (SCID) mouse, it became possible to transplant human tumours into the mouse, creating xenograft models. Such models can be used to rapidly assess tumour tissue and cell lines *in vivo* but they do not fully recapitulate the behaviour of an endogenous tumour because many features of the tumour microenvironment, such as stromal cells, vasculature and immune cells, are missing. The tumour xenograft is also likely to be less heterogeneous than the endogenous tumour because cells in culture are under high selective pressure. These factors have contributed to the limited success of xenograft models in drug development (for a review, see [144]).

Many inbred strains that spontaneously develop cancer at high frequency have been established, and these, as well as mice that have been treated with a mutagen, are useful for studying the properties of endogenous cancers *in vivo*. They have been used to identify cancer genes and to assess the effects of carcinogens and therapeutic compounds. However, these models may be biased towards specific types of tumour that show variable penetrance and latency and that do not accurately reflect common human cancers [143].

### 2.2 Genetically engineered mouse models

Genetically engineered mouse models represent a major advance in cancer research that allows for the study of gene function *in vivo* and for the creation of models that more accurately recapitulate human cancers. Genetically engineered models can be classified as transgenic or endogenous [143].

#### 2.2.1 Transgenic models

Transgenic mice can be created to study the effect of overexpressing an oncogene or a dominant-negative tumour suppressor gene, which encodes a mutant tumour suppressor that can inactivate the wildtype protein. Transgenic mice can be generated by pronuclear microinjection, in which a construct containing the gene of interest (transgene) is microinjected into the mouse oocyte after fertilisation and randomly integrates into the genome, usually in tandem copies. If the transgenic cells contribute to the germ line, the genetic change can be transmitted to the next generation, producing mice that are fully transgenic and establishing a strain. Many genes involved in cancer development are also essential for mouse development. Therefore, to prevent embryonic lethality and to restrict overexpression to specific tissues, the construct containing the gene of interest also contains promoter elements designed for spatial and temporal restriction of gene expression. For example, the Tet-On and Tet-Off systems [145] promote gene expression in the presence or absence of doxycycline, a non-toxic analogue of tetracycline, while fusing the gene of interest to a gene encoding the oestrogen receptor binding domain results in an inactive protein that is activated upon treatment with Tamoxifen [146].

Limitations of the microinjection method include the possibility that, because the transgene integrates randomly, it could disrupt other genes, resulting in a phenotype that does not reflect the function of the gene of interest (for a review, see [147]). In addition, the tendency of the transgene to integrate in multiple copies could result in excessive overexpression that is toxic to the animal [147]. However, transgenic mice have made a significant contribution to cancer research. In the earliest examples, mouse models were used to demonstrate the role of oncogenes in cancer. For example, tissue-specific overexpression of the *Myc* oncogene in mammary glands and B-cells resulted in the generation of mice prone to breast cancer [148] and lymphomas [149], respectively. Overexpression of dominant-negative mutant tumour suppressor genes has also proved effective, e.g. type II transforming growth factor beta (*Tgfβ*) receptor has been shown to accelerate chemically induced tumourigenesis in the mammary gland and lung [150].

#### 2.2.2 Targeted/endogenous models

A knockout mouse can be created to study the effect of inactivating a tumour suppressor gene. In this method, a targeting vector is transfected into embryonic stem (ES) cells, which are harvested from the inner cell mass of mouse blastocysts. The vector must share homology with the region of the mouse gene that is being targeted, i.e. the tumour suppressor gene of interest, and must also contain genes for selection, such that only cells in which the vector DNA has replaced the endogenous DNA by homologous recombination will survive. The surviving ES cells are injected back into a blastocyst, and will contribute to all cell lineages, including the germ line [151]. The targeting vector can be engineered to knock out the whole gene or part of a gene, or small changes can be introduced into the gene sequence. Alternatively, the complete gene under the control of a strong promoter can be introduced to create a knockin mouse for overexpressing oncogenes. By targeting a single copy to the genome, this overcomes the problems associated with pronuclear microinjection (for a review, see [147]).

As with transgenic mice, mutations can be spatiotemporally regulated. Conditional mouse models frequently use the Cre–lox system from bacteriophage P1, in which Cre recombinase catalyses recombination between loxP sites [152], and the intervening DNA is deleted or inverted, depending on the orientation of the sites [153]. loxP sites can therefore be placed on either side of a gene region to remove that region in the presence of Cre. Large-scale chromosomal deletions and inversions can also be generated by placing loxP sites, either in the same orientation for deletions or the reverse orientation for inversions, further apart on the chromosome [154,155], while chromosomal translocations can be created by placing a loxP site at each breakpoint [156] on non-homologous chromosomes. Conditional oncogene expression can be achieved by inserting a stop cassette, which is flanked by loxP sites, between the promoter and the first exon such that Cre-mediated excision of the cassette results in expression of the gene [157,158].

Unlike the conditional expression systems in transgenic mice, once Cre recombinase has been expressed, the change is irreversible, and there is evidence to suggest that Cre can be cytotoxic, perhaps due to recombination at pseudo-loxP sites (see [159]). In addition, the Cre–lox system cannot generate conditional point mutations, and this represents a significant limitation since point mutations and deletions do not always produce the same phenotype [143]. However, the Cre–lox system has proved invaluable in creating models that would otherwise not arise or survive. For example, homozygous *Brca1* and *Brca2* knockouts die early in embryogenesis, and heterozygous mice are not tumour-prone, but mice harbouring a Cre-mediated deletion of *Brca1* [160] or *Brca2* and *Trp53* [161] in the adult mammary gland do develop mammary tumours. Likewise, *Trp53* mutations have been identified in many types of human cancer, but if *Trp53* is mutated in all cells, the mouse is most likely to develop lymphomas and sarcomas. Conditional *Trp53* mutations can be used to create models for human cancers that are driven by *Trp53* mutation in other tissues [159]. The Flp/FRT system from *Saccharomyces cerevisiae* is an alternative to Cre–lox that works in a similar way (see [159]).

### 2.3 Mouse models in cancer gene discovery

The methods described earlier in the review can also be applied to the identification of candidate cancer genes in the mouse. For example, array CGH has been used to identify regions of copy number change in mouse models of malignant melanoma [162] and pancreatic islet carcinomas [163]. However, as with human cancers, by the time the cancer has presented, it is difficult to distinguish the important driver mutations from the background of passenger mutations.

The genetically engineered mouse models discussed thus far are useful for studying the function of a particular gene or for representing a specific human cancer, but the tumours in these models do not evolve naturally. In general, the initiating event, i.e. the engineered mutation, is present throughout a tissue, whereas in natural tumourigenesis, the tumour develops from one mutated cell. Likewise, in mouse models used to study the combined action of multiple genes in cancer, the genes of interest are usually simultaneously mutated, whereas “natural” tumours progress through a multi-step process, where mutations are gradually acquired. Finally, many mouse models are designed to show high penetrance and short latency to keep costs down, but as a result they may not possess many of the co-operating oncogenic events that would eventually be acquired by a naturally evolving tumour (for reviews, see [143,144]).

It is important that the mutations in mouse models used to identify novel cancer genes reflect the mutations found in human cancers, and this requires more accurate modelling of the natural evolution of tumours.

### 2.4 Forward genetic screens in the mouse

Forward genetic screens using somatic mutagens are a powerful approach for cancer gene discovery in the mouse. Insertional mutagens allow for relatively unbiased, genome-wide, identification of both novel cancer genes and collaborations between genes involved in cancer. Chemical mutagenesis, using agents such as *N*-ethyl-*N*-nitrosourea (ENU), is a highly efficient way of inducing tumours in mice but the causal mutations can be hard to identify. In contrast, insertional mutagenesis using retroviruses and transposons is an effective approach for inducing the stepwise progression of a cell to malignancy, and for the identification of the causal genetic lesions, because the mutagen acts as a molecular ‘tag’ allowing its location in the genome to be easily determined.

#### 2.4.1 Retroviral insertional mutagenesis

##### 2.4.1.1 Mechanisms of mutagenesis

The slow transforming retroviruses murine leukaemia virus (MuLV) and mouse mammary tumour virus (MMTV) have been widely used for insertional mutagenesis in the mouse. Unlike acute transforming retroviruses, which induce tumours by expression of a viral oncogene, slow transforming retroviruses do not carry an oncogene, and tumours are induced by mutations caused by insertion of the retrovirus into the host genome. Consequently, tumours develop with a longer latency of 3–12 months, compared with 2–3 months for acute transforming retroviruses [164]. MMTV was identified as a causative agent in several strains of mice that were prone to mammary tumours, while MuLV was identified as a causative agent in the lymphoma-prone AKR mice (see [165]).

Retroviruses infect host cells by binding of the viral envelope proteins to cell surface receptors. Once the retrovirus has inserted into the host genome, forming a provirus, it will produce viral envelope proteins that occupy the cell surface receptors and prevent reinfection of the same cell. However, recombination with endogenous viral sequences results in the production of envelope proteins that bind to other receptors. This, combined with the fact that many proviruses have defective envelope coding sequences, enables retroviruses to reinfect the same cell, resulting in the accumulation of mutations. Mutations that confer a growth advantage on the cell co-operate in tumour formation, and the process therefore recapitulates the multi-step progression of human tumours (for reviews, see [164,166]).

**Fig. 2:**
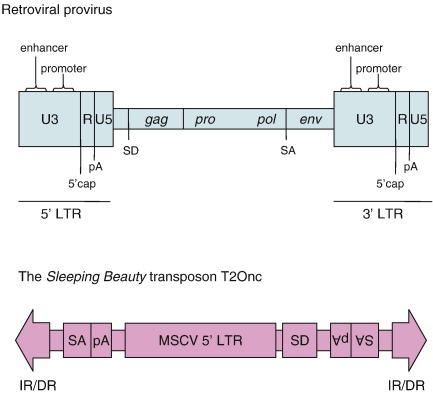
Structure of insertional mutagens used for cancer gene discovery in the mouse. (A) The provirus contains two long terminal repeats (LTRs) flanking the genes required for viral assembly. Elements within the LTRs drive transcription of the viral genes but can also induce mutation of nearby cellular genes. Splicing of a viral splice donor (SD) or cryptic splice donor (not shown) to a splice acceptor or cryptic splice acceptor in the first intron or 5′ UTR of a cellular gene results in the formation of a chimeric transcript, in which the celluar gene is coupled to the viral promoter. Splicing of a splice donor or cryptic splice donor in a cellular gene to a viral splice acceptor (SA) or cryptic splice acceptor (not shown) can cause premature termination of gene transcription owing to the presence of polyadenylation signal (pA) and cryptic polyadenylation signals (not shown) in the LTR. Adapted from figure in Uren et al. (see [164,168]). Figure is not to scale. (B) Structure of the *Sleeping Beauty* transposon T2Onc [242]. The presence of splice acceptors (SA) and polyadenylation signals (pA) in both orientations enables premature termination of gene transcription from intragenic insertions in both orientations. The transposon also contains the murine stem cell virus (MSCV) 5′ LTR and a splice donor (SD) site that can induce promoter mutations in cellular genes. Elements for mutagenesis are flanked by 2 IR/DR elements, shown as arrows, which are required for transposon mobilisation.

The MuLV provirus consists of viral genes flanked by two long terminal repeats (LTRs), which are composed of three parts: U3, R and U5 [164]. Elements within the LTRs drive expression of the viral genes but can also disrupt host genes. U3 contains enhancer and promoter sequences, while R contains transcription start and termination sites. High levels of viral transcription and, therefore, host gene disruption, will only occur in cells containing transcription factors that bind to U3. The propensity of MuLV to induce T- and B-cell lymphomas can be attributed to its dependence upon T- and B-cell-specific transcription factors, including *Runx*, *Ets* and *Myb* (see [167], Fig. 2). MMTV, and indeed other retroviruses, have a similar structure to MuLV.

**Fig. 3:**
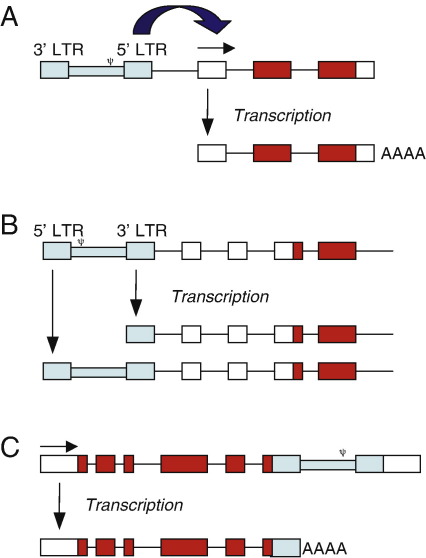
Mechanisms of mutagenesis by the murine leukaemia virus. The provirus is shown in blue; coding and non-coding exons are shown in red and white, respectively. (A) Enhancer mutation. An enhancer element in the 5′ LTR of MuLV can cause upregulation of nearby cellular genes. Oncogenic insertions of this type are most frequently found upstream and in the antisense orientation with respect to the cellular gene(s) that they are mutating. (B) Promoter mutation. Insertion of MuLV into the promoter region of a cellular gene results in chimeric transcripts that are produced at higher levels than the endogenous gene transcript. (C) Truncating mutation. Intragenic MuLV insertions can cause premature termination of gene transcription, resulting in either gene upregulation or gene inactivation. The figure shows an insertion within the 3′ UTR region, which may remove mRNA-destabilising motifs, thereby stabilising the gene transcript.

Retroviruses can mutate host genes in a number of different ways (Fig. 3). The most common mechanism is enhancer mutation, where one of the U3 enhancers upregulates expression of host genes, which may be some distance away from the retroviral insertion. Most proviruses causing enhancer mutations are found upstream of the mutated gene in the antisense orientation or downstream in the sense orientation. Several possible explanations for this are that upregulation of the host gene may be impeded if the viral promoter intercepts the viral enhancer and host gene, and that viral enhancers may only be functional if they are not transcribed (see [164,168]). *Myc* and *Gfi1* are frequent targets of enhancer mutation in retroviral insertional mutagenesis [169–171]. *Myc* is mutated in many types of human cancer and encodes a transcription factor thought to regulate the expression of 15% of all genes, including those involved in cell division, cell growth and apoptosis (see [172]). *Gfi1* is a zinc finger transcriptional repressor that is involved in cell fate determination and differentiation, including in T- and B-cells [173,174].

An alternative mechanism of mutagenesis is the promoter mutation, where the retrovirus inserts in the sense orientation into the promoter region of a host gene. This uncouples the host gene from its own promoter and places it under the control of the viral promoters, resulting in the production of elevated levels of the wildtype protein from chimeric transcripts comprising part of the viral sequence and the complete coding region of the host gene [175]. Promoter mutations led to identification of *Evi1* as a potential oncogene [176–178]. *EVI1* encodes a zinc finger transcription factor that is frequently overexpressed in myeloid malignancies. It is involved in several recurrent rearrangements, including 2 translocations that result in the fusion transcripts *AML1/MDS1/EVI1* and *ETV6/MDS1/EVI1*, where *MDS1* and *EVI1* are also expressed as a readthrough transcript in normal tissues (for a review, see [179]).

Since the retrovirus contains a polyadenylation signal within the R region of the LTR and a cryptic polyadenylation signal in the antisense orientation intragenic retroviral insertions in both orientations can cause premature termination of gene transcription. Insertions within the 3′ UTR that truncate a transcript such that mRNA-destabilising motifs are removed will give rise to a more stable transcript and, as a result, increased levels of the wildtype protein (see [164]). The oncogenes *Pim1* and *Mycn* are frequently mutated in this way [180–182]. *Pim1* encodes a serine/threonine kinase that is frequently overexpressed in human prostate cancer [183], while *Mycn* encodes a transcription factor related to *Myc* that is amplified in a variety of human tumours, most notably neuroblastomas [184,185].

Intragenic insertions can also activate a gene by causing C-terminal or N-terminal truncation of the encoded protein. Insertions in oncogenes *Myb* and *Notch1* cause both N-terminal and C-terminal truncations [164,186]. C-terminally truncated Notch1 lacks the destabilising PEST domain and is therefore produced at increased levels, while N-terminal truncations remove the extracellular domain, resulting in a constitutively active intracellular domain expressed from the viral promoter or from a cryptic promoter in *Notch1* [187]. Activating mutations within the extracellular and PEST domains of NOTCH1 have been observed in human T-cell acute lymphoblastic leukaemia [188], in which NOTCH1 plays an important role. Thus analysis of the distribution of insertions within an oncogene may therefore help to explain how the gene is mutated in human cancer.

Intragenic insertions may also cause gene inactivation, either through premature termination of transcription or by disrupting gene splicing (see [164]). It is therefore possible to identify tumour suppressor genes by retroviral insertional mutagenesis, although they are found much less frequently than oncogenes because both copies of the gene must be inactivated. Mutation at the *Nf1* locus is observed in acute myeloid leukaemia in BXH2 mice [189], which contain MuLV insertions [190], while in an insertional mutagenesis screen of *Blm*-deficient mice, 11 genes met the criteria for tumour suppressor genes, including *Rbl1* and *Rbl2*, which are paralogues of *Rb1* [191]. *Blm*-deficient mice have a mutation in the RecQ protein-like-3 helicase gene [192] and show a predisposition to cancer due to increased frequencies of mitotic recombination [193]. There is an increased likelihood of finding tumour suppressor genes in these mice because they have a higher probability of a normal allele being lost so that only one insertion is required to inactivate the gene [193]. However, candidate tumour suppressor genes still only accounted for 5% of all genes identified in the screen by Suzuki et al. [191]. In theory, insertional mutagenesis screens should have a better chance of finding haploinsufficient tumour suppressor genes, but none have yet been unambiguously identified [164].

Insertional bias could also account for the paucity of tumour suppressor genes identified in retroviral screens. For example, MuLV shows a strong preference for integration near to the transcription start sites of actively transcribed genes [194] and is therefore less likely to disrupt a gene by intragenic insertion. However, it is possible that promoter mutations could also cause gene inactivation, as CpG islands in the retroviral LTRs are methylation targets, and DNA methylation could “spread” to CpG islands in the host gene, resulting in gene silencing (see [195]). Retroviruses prefer to insert into open chromatin [196,197], but different retroviruses show different target site preferences, suggesting that virus-specific interactions are involved [198]. DNA sequence does not seem to influence target site selection [199]. The tendency for MuLV to insert in the promoter region suggests that it interacts with cellular proteins bound near start sites [194,198].

##### 2.4.1.2 Identifying candidate cancer genes

**Fig. 4:**
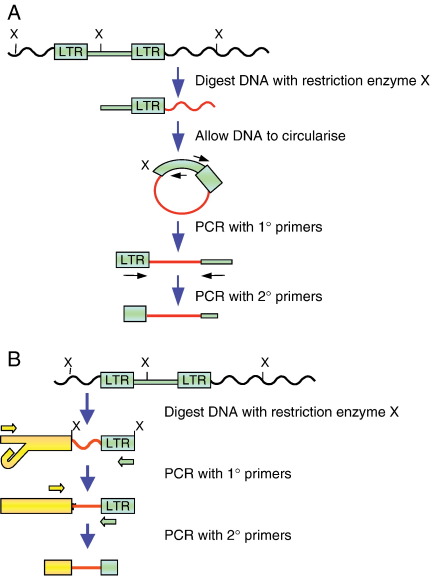
Isolation of retroviral insertion sites. (A) Inverse PCR. Tumour DNA is digested using restriction enzyme X and the restricted DNA is allowed to circularise. Genomic DNA flanking retroviral insertions are amplified using PCR primers that bind within the insertion and point out towards the genomic DNA. A second round of PCR is performed using nested primers. The amplified DNA is sequenced and mapped to the mouse reference genome. (B) Splinkerette PCR. As for inverse PCR, except that instead of circularising the digested DNA, a splinkerette adapter (shown in yellow) is ligated and genomic DNA flanking the retroviral insertions is amplified using PCR primers that bind to the adapter and the retroviral LTR.

The retroviral insertions act as ‘tags’ for identifying the mouse genes that are mutated by insertional mutagenesis, and sequencing of the mouse genome and the development of high-throughput genomic techniques have made it possible to identify thousands of insertions in a single screen. Insertion sites were initially identified using methods that involve Southern blot analysis and genomic library screening, followed by genome walking to find the mutated gene (see [164,167]). However, these have been replaced by PCR-based methods, in which mouse genomic DNA flanking the insertion sites is amplified and is then mapped back to the genome. One such method, known as viral insertion site amplification (VISA) involves using a PCR primer designed to bind to the MuLV LTR and a degenerate, restriction-site specific primer that enables amplification of the DNA between the insertion and a nearby restriction site [200,201]. In inverse PCR and linker-mediated PCR-based methods (Fig. 4) the genomic DNA is digested with a restriction enzyme prior to PCR amplification. In inverse PCR, the digested genomic DNA is allowed to ligate to itself to form a circular template. PCR primers bind to the retroviral DNA and point out towards the genomic sequence, resulting in amplification of genomic DNA directly flanking the retrovirus [202,203]. Only DNA fragments of a suitable length for efficient circularisation and for PCR amplification will be detected [164].

In linker-mediated PCR, rather than the digested DNA ligating to itself, it is ligated to a linker, and this enables shorter insertions to be identified. One primer is designed to bind to the linker, and the other binds to the retroviral sequence. A number of methods have been developed, each with a different approach for avoiding amplification of DNA that has linkers at both ends but no retroviral DNA. Vectorette PCR involves the use of a double-stranded linker with a cohesive end for ligation to restricted DNA and a central region with a mismatch [204]. The primer is the same sequence as the mismatched part of the upper strand, and this prevents initiation of priming from the vectorette until the complementary strand has been synthesised by priming from within the retroviral insertion. However, this method suffers from non-specific annealing of the primers and ‘end-repair’ priming, in which the ends of unligated vectorettes initiate priming and enable PCR amplification without involving the retroviral-specific primer (see [205]). Any errors that cause amplification of DNA that is not flanking an insertion will lead to the false identification of insertion sites.

An improved method uses splinkerettes, which incorporate a hairpin structure on the bottom strand, rather than a mismatch sequence [205]. The primer has the same sequence as the upper strand and, as with vectorette PCR, cannot anneal until the complementary strand has been synthesised. The stable hairpin does not enable end-repair priming and only the upper strand can act as a non-specific primer. In all the PCR-based methods, insertions are only identified if target sites for the chosen restriction endonuclease are close enough to the insertion for the intervening region to be amplified. Coverage can be improved by using multiple restriction endonucleases [164].

Once the insertion-flanking genomic DNA has been amplified, the PCR products must be separated for sequencing. In the past, products were separated using agarose or polyacrylamide gels, but rare insertions are likely to be missed, and gel extraction is painstaking and subjective. An alternative method is to subclone the PCR products directly into a vector. By shotgun cloning the total mixture, it is possible to maintain the relative proportions of insertions from the starting material. However, it also means that more sequencing will be required to capture the rare insertions (see [164]). The VISA approach sequences PCR products directly, without subcloning, which reduces the risk of sequencing contaminating products [201]. The latest method uses massively parallel sequencing technology from 454 Life Sciences (http://www.454.com) [206,207], in which fragmented genomic DNA is ligated to short adaptors that are used for purification, amplification and sequencing. The DNA is denatured and immobilised onto beads, where PCR amplification and sequencing occur. This approach is extremely high-throughput, does not rely on cloning and is capable of detecting rare insertions. However, it can encounter problems when dealing with repetitive regions and long runs of a single nucleotide.

The next step is to map the sequenced DNA to the genome using a DNA alignment algorithm. For large screens, it is an advantage to be able to find high quality alignments quickly [164]. The Sequence Search and Alignment by Hashing Algorithm (SSAHA) (SSAHA2, [208]) converts the genome into a hash table, which can then be rapidly searched for matches. Sequences in the database (the mouse genome) are preprocessed into consecutive *k*-tuples of *k* contiguous bases and the hash table stores the position of each occurrence of each *k*-tuple. The query sequence (sequenced DNA) is also split into *k*-tuples and the locations of all occurrences of these sequences in the database, i.e. the “hits”, are extracted from the hash table. The list of hits is sorted, and the algorithm searches for runs of hits in the database that match those in the query sequence. Having identified regions of high similarity, sequences are fully aligned using cross_match [209], which is based on the Smith–Waterman–Gotoh alignment algorithm [210,211]. Because the database is hashed, search time in SSAHA2 is independent of database size, provided *k* is not too small. SSAHA2 is therefore three to four orders of magnitude faster than the BLAST alignment algorithm [212], which scans the database and therefore performs at a speed that is directly related to database size [208].

If the PCR mixture has been shotgun cloned and preferably sequenced to a high depth, there may be more than 1 read per insertion site. Reads from a single tumour that map to the same genomic region must therefore be clustered into single insertion sites. Like the mutations in human cancer, tumour DNA will contain both insertions that drive oncogenesis (oncogenic insertions) and insertions that are passengers (background insertions). In theory, most identified insertions should be oncogenic because these, and particularly the earliest events in tumourigenesis, should be present in most, if not all, tumour cells, whereas background insertions should be present in a smaller proportion of cells. However, background insertions that occur early in tumour development in a cell containing oncogenic insertions could also be highly represented in the final tumour (see [213]).

Clustering of insertions from different tumours into common insertion sites (CISs) helps to distinguish oncogenic and background insertions. In theory, background insertions should be randomly distributed across the genome. Therefore, for small-scale screens, a gene in the vicinity of a cluster of insertion sites in different tumours is a strong candidate for a role in cancer. Methods for identifying statistically significant CISs, i.e. regions that are mutated by insertions in significantly more tumours than expected by chance, have involved generating a random distribution of insertions across the genome and obtaining an estimate of the number of false CISs in windows of fixed size using Monte Carlo simulation [214] or the Poisson distribution [175]. These methods can be used to define the maximum window size in which insertions must fall to be considered non-randomly distributed. However, for larger scale screens, the window must be decreased to a size that is smaller than the spread of insertions within a single CIS so that many CIS are missed [213]. In addition, the above methods assume that insertions are randomly distributed and take no account of insertional biases [215].

A more recent approach for CIS detection overcomes these problems by using a kernel convolution (KC) framework, which calculates a smoothed density distribution of inserts across the genome [213]. The scale (kernel size) can be varied so that CISs of varying widths can be identified. Decreasing the kernel size may identify separate CISs affecting the same gene, while increasing the kernel size will identify CISs where insertions are widely distributed in or around a gene. The method can be used for large-scale studies because it keeps control of the probability of detecting false CISs. The threshold for significant CISs is based on the alpha-level defined by the user and on a null-distribution of insertion densities obtained by performing random permutations. A background distribution, such as the location of transcription start sites, can be provided to correct for insertional biases [213].

The final step is to identify the genes that are being mutated by insertions within CISs. This may be relatively straightforward for intragenic insertions, but for enhancer mutations, which may have long distance effects, it is often difficult to identify the mutated gene unequivocally. Measuring the expression and transcript size of candidate genes in insertion-containing tumours can shed some light, but animal models and analysis of the orthologues in human cancer data are required for more conclusive evidence [164].

A number of screens have been performed in recent years that have each identified hundreds of insertion sites [175,191,201,214,216–223]. The results of many screens have been collated and stored in the Retroviral Tagged Cancer Gene Database (RTCGD; http://rtcgd.abcc.ncifcrf.gov/) [171]. At the time of writing, the database contains CISs associated with 540 genes from 30 screens (database accessed December 2008). Users can search for individual genes of interest, or for CISs identified using particular mouse models and/or in particular tumour types. Genes with the most CISs are *Gfi1* and *Myc*, with 82 and 78 insertions across all screens, respectively.

##### 2.4.1.3 Identifying co-operating cancer genes

Retroviral insertional mutagenesis is a powerful tool for identifying genes that collaborate in tumour development. Collaborations can be identified by analysing the co-occurrence of CISs in individual tumours. For example, proviral activation of *Meis1* and *Hoxa7* or *Hoxa9* is strongly correlated in myeloid leukaemias from BXH2 mice [190,224]. *Meis1* and *Hoxa9* are targets of translocation in human pre-B leukaemia [225] and acute myeloid leukaemia (AML) [226], respectively, and they are frequently co-expressed in human AML [227]. Both genes encode homeodomain transcription factors that bind to Pbx, and Meis1–Pbx and Hox–Pbx complexes have been shown to co-occupy the promoters of leukaemia-associated genes, such as *Flt3* [228].

A two-dimensional Gaussian Kernel Convolution method has recently been developed for identifying co-operating mutations in insertional mutagenesis data [229]. It is based on the Kernel Convolution framework used for identifying CISs [213]. The method has been applied to the data in RTCGD and, as well as finding previously characterised interactions, such as *Meis1* and *Hoxa9/Hoxa7*, it also finds novel interactions, such as *Rasgrp1* and *Cebpb*, which are both known to play a role in *Ras*-induced oncogenesis [229]. However, as retroviral-induced tumours are oligoclonal, it is difficult to prove that tagged genes are in the same cell, and therefore that they collaborate [230]. In an alternative approach, retroviral screens are performed on transgenic mice overexpressing known oncogenes, and knockout mice harbouring inactivated tumour suppressor genes, to identify genes that collaborate with the overexpression of oncogenes, and loss of tumour suppressor genes, respectively. For example, 35% of B-cell lymphomas generated in MuLV-infected *EμMyc* transgenic mice, in which *Myc* is overexpressed in B-cell progenitors under the control of the immunoglobulin heavy chain enhancer, have an insertion in *Pim1* or the polycomb group protein *Bmi1* [231]. Bmi1 collaborates with Myc by inhibiting *Ink4a/Arf*, and therefore inhibiting Myc-induced apoptosis [232]. In concurrence with these findings, *Myc* insertions were identified in 20% of tumours from MuLV-infected *Cdkn2a* (*Ink4a/Arf*)-deficient mice, but none contained insertions in *Bmi1* [218]. Insertional mutagenesis also identifies genes that can functionally complement one another in tumour development. For example, in MuLV-infected *EμMyc* mice, activation of *Pim2* increases from 15% to 80% in compound mutant mice lacking *Pim1* expression [233], while *Pim3* is selectively activated in mice lacking *Pim1* and *Pim2* expression [175]. Pim1 is a coactivator of Myc that is required for expression of around 20% of all Myc target genes [234]. Pim kinases also appear to suppress Myc-induced apoptosis, but it is not clear whether this mechanism or Myc coactivation is responsible for the co-occurrence of *Pim1* and *Myc* mutations observed in lymphomagenesis (for a review, see [235]). *Pim1* also collaborates with *Myc* in human prostate cancers [236].

Retroviral screening of a mouse model for human myeloid leukaemia has identified 6 CIS genes, including *Plag1* and *Plagl2*, which co-operate with the oncogenic fusion gene *CBFB-MYH11* [237]. This screen used a replication-defective retrovirus, cloned amphotropic virus 4070A, to limit the number of mutations and therefore to show that mutation of only one or a few genes were sufficient to induce tumorigenesis. Other studies using replication-competent viruses report 3–6 insertions in a single tumour [175,214] but, as mentioned above, retroviral-induced tumours are oligoclonal and it is therefore difficult to make a reliable estimate of the number of insertions in a tumour clone (see [167]).

##### 2.4.1.4 Generating tumours of different types

As discussed previously the dependence of retroviruses on cell-type-specific transcription factors limits the range of tumours that they can induce. There have been some successful attempts to alter the propensity of MuLV for T-cell lymphomas by using an *EμMyc* transgenic mouse, which results in predominantly B-cell lymphomas [231], and by expressing platelet derived growth factor B-chain (*PDGFβ*) from an MuLV-based retrovirus to generate mice with glioblastomas, which require activation of PDGF receptors for tumourigenesis [219]. Mutations in the retroviral LTR may also lead to a change in tumour type, but manipulated viruses have a tendency to revert to wildtype [164]. In addition, MuLV and other retroviruses cannot infect nondividing cells, and infection is inefficient in slowly replicating cells and in tissues that have a basement membrane or mucin layer [238,239]. Transposon insertional mutagenesis is an alternative method that provides the possibility of generating a wider spectrum of tumours.

#### 2.4.2 Transposon-mediated insertional mutagenesis

Like retroviruses, transposons are genetic elements that can mobilise within the genome. They are classified according to their mechanism of transposition. DNA transposons move by a “cut and paste” mechanism, in which they are excised from one site in the genome and integrated into another. Retrotransposons transpose via an RNA intermediate and are classified into LTR retrotransposons, which encode reverse transcriptase and transpose in a similar manner to retroviruses, and non-LTR retrotransposons, which are transcribed by host RNA polymerases and may or may not encode reverse transcriptase [240].

##### 2.4.2.1 The *Sleeping Beauty* transposon system

While DNA transposons are actively mobile in plants and invertebrates, all of the elements that have been so far identified in vertebrates are non-functional [164]. However, they can be mobilised in the mouse by using an invertebrate DNA transposon or by reconstructing a degenerate vertebrate transposon. *Sleeping Beauty* (SB) is a synthetic transposon derived from dormant DNA transposons of the Tc1/Mariner family in the genomes of salmonid fish. An active transposon, named SB10, was synthesised by directed mutagenesis on the basis of a consensus sequence obtained by aligning 12 degenerate transposon sequences from 8 species [241]. SB consists of two inverted repeat/direct repeat (IR/DR) elements of ∼ 230 bp each, flanking a cargo sequence [242]. Transposition occurs via binding of a transposase enzyme to two sites in each IR/DR [243]. All four binding sites are required for transposition and, in general, the closer the IR/DRs, the higher the transposition efficiency [243]. Higher levels of transposition have been achieved by introducing point mutations into the transposase, producing, for example, the SB11 [244] and SB12 [245] transposases.

The utility of SB for oncogenic insertional mutagenesis was first demonstrated in two studies published in 2005 [246,247]. In both studies, transposons were introduced into mice by pronuclear injection of a linear plasmid containing one copy of the transposon, which forms a multicopy concatemer of variable length at a single site in the mouse genome. SB was mobilised by crossing these mice to mice expressing a transposase from a ubiquitous promoter. Collier et al. [246] used a transgene containing the SB10 transposase under the control of the CAGGS promoter to mobilise around 25 T2/Onc transposons, while Dupuy et al. [247] used the more active SB11 version knocked into the endogenous *Rosa26* locus to mobilise 150–350 copies of the T2/Onc2 transposon. T2/Onc and T2/Onc2 were engineered to contain elements for mutagenesis much like those in retroviruses (Fig. 2). The cargo of both transposons contains the 5′ LTR of the murine stem cell virus (MSCV) followed by a splice donor, as well as splice acceptors followed by polyadenylation sites in both orientations. The transposons are therefore capable of disrupting genes by promoter mutation, N-terminal and C-terminal truncation and gene inactivation but, unlike retroviruses, they show low enhancer activity [247]. T2/Onc and T2/Onc2 are essentially the same, except that T2/Onc2 contains a larger fragment of the *Engrailed* splice acceptor and the IR/DRs have been optimised for transposase binding [247]. In the study by Dupuy et al. [247], there was a high rate of embryonic lethality and of the 24 T2/Onc2;Rosa26SB11 mice that survived to weaning, all developed cancer, most commonly T-cell lymphomas but also other haematopoietic malignancies and, in a few cases, medulloblastomas and intestinal and pituitary neoplasias. Some mice had 2 or 3 types of cancer and all died within 17 weeks. In contrast, in the study by Collier et al. [246], mice on a wildtype background did not develop tumours, but those on an *Arf*-null background developed sarcomas at an accelerated rate. The difference between the two studies most likely reflects the differences in transposon copy number and in transposase expression and activity [248]. Transposase expression in CAGGS-SB10 mice has since been shown to be low and variegated in most tissues, probably due to epigenetic silencing of the transgene, while transposase expression is high in nearly all cell types in Rosa26SB11 mice [248]. However, transposase is expressed in the testes of CAGGS-SB10 mice, which show high rates of transposition in the male germline [248,249].

Transposons, like retroviruses, can be used to identify co-operating cancer genes. For example, *Braf* was frequently mutated in *Arf*-null mice, suggesting that these genes co-operate in tumour formation [246], while of the six T-cell tumours containing *Notch1* mutations, three also contained insertions mutating *Rasgrp1*, and 2 of these contains *Sox8* mutations, suggesting that these three genes also co-operate [247].

While a number of the genes identified in the haematopoietic malignancies of T2/Onc2;Rosa26SB11 mice had been previously identified in retroviral mutagenesis, other genes had not [247]. This indicates that transposon mutagenesis is a complementary approach for cancer gene discovery, and may reflect differences in insertional bias. While MuLV shows a strong preference for inserting near transcription start sites [194], SB shows a less pronounced preference and shows no preference for actively transcribed genes [250]. SB inserts at TA dinucleotides and therefore shows a bias towards AT-rich sites, particularly those with the consensus sequence ANNTANNT [251,252]. However, most significant is the strong tendency of SB to transpose to sites close to the concatemer. This phenomenon, known as “local hopping”, results in a non-random distribution of insertions that hampers CIS detection. Another potential hindrance to cancer gene identification is the ability of transposons to excise themselves and reinsert multiple times. SB leaves a small footprint upon excision, and it is possible that, at least in exons, this could continue to cause gene disruption that would not be identifiable [248]. Likewise, the excision in some cells of transposons that had been critical for tumour development could result in a more heterogeneous tumour in which cancer gene identification would be more complicated. However, it is possible that such an event would be deleterious and that the cell would be eliminated [248] and, as SB transposition efficiency is higher for methylated [253] and heterochromatic [254] transposons, excision of transposons involved in gene disruption may be relatively rare. A further drawback of SB, and possibly other DNA transposons, is that transposition may induce genomic rearrangements, including deletions and inversions near to the transposon concatemer, and tumourigenesis could therefore be initiated by genes disrupted by these rearrangements rather than by mobilised transposons [255].

One of the key benefits of using a transposon such as SB for insertional mutagenesis is that the mutagenic elements can be modified to control the types of mutation that occur. For example, modifying the cargo to enable only truncating mutations could increase the likelihood of identifying tumour suppressor genes [248]. Tissue-specific promoters can be integrated as cargo, making transposons an attractive mutagen for cancer gene discovery in a range of cancer types [256]. Spatial and temporal transposition could also be achieved by introducing a lox–stop–lox cassette between the SB transposase promoter and cDNA, such that transposition is induced upon the addition of Cre [256].

Identification of cancer genes in SB mutagenesis follows much the same procedure as for retroviruses. Largaespada and Collier [257] have developed a technique that uses linker-mediated PCR but that enables PCR amplification of DNA flanking both sides of the transposon to maximise coverage. Primers were designed to bind to the IR/DR sites and to synthetic adapters. Unlike in retroviral mutagenesis, tumour cells contain a concatemer of non-transposed elements. To avoid repeated cloning of the junctions between these elements, “blocking” primers can be used that bind to the plasmid DNA flanking each transposon in the concatemer but that have blocked 3′ ends to prevent polymerase extension. Alternatively, after linker ligation, the DNA could be redigested with an endonuclease that cuts within the flanking plasmid DNA so that the primer binding sites are separated on different molecules (see [257]).

##### 2.4.2.2 Alternative mutagens for transposon insertional mutagenesis

The active invertebrate transposons *piggyBac* and *Minos* are the only other DNA transposons that have so far been mobilised in the mouse [248]. The *piggyBac* transposon, isolated from the cabbage looper moth (*Trichoplusia*), mobilises in mouse somatic cells and in the germline, and it can carry a larger cargo than SB [258]. The coding sequence of *piggyBac* has been codon-optimised to enable higher levels of transposition in the mouse, and inducible versions have been generated by fusing the transposon to the ERt^2^ oestrogen receptor ligand-binding domain [259]. Unlike SB, it shows a strong preference for inserting into genes in the mouse [258] and in human cell lines [260]. The *Minos* transposon, from *Drosophila hydei*, has attracted interest because it shows a low insertional bias and high transposition efficiency in a range of animals (for a review, see [261]). However, it has so far shown only weak *in vivo* activity in the mouse [262,263].

Retrotransposons are also gaining attention as potential insertional mutagens. Long interspersed nuclear elements (LINEs) are non-LTR retrotransposons that are transcribed into mRNA by RNA polymerase II and encode two proteins that are essential for transposition [264]: a protein that binds to single-stranded RNA [265] and a protein with reverse transcriptase and endonuclease activity [266,267]. 17% of the human genome is composed of LINE-1 (L1) elements [268]. Transcription of endogenous L1 elements is generally inefficient but there are a small number of highly active “hot L1s”, which were used to generate a transgenic mouse model of L1 retrotransposition that showed a higher frequency of de novo somatic L1 insertions [269]. A 200-fold increase in transposition in the mouse germline has also been achieved by codon optimisation of the human L1 coding region [270]. L1 mobilises by a “copy and paste” mechanism. It is therefore an attractive mutagen for forward genetic screens because, unlike DNA transposons, it is capable of self-expansion and the original insertion remains intact, aiding identification of mutated genes [248,271]. In addition, it appears to show no [272], or only a slight insertion site preference [269], for inserting into genes and there is no local hopping because the RNA intermediate must exit and re-enter the nucleus before inserting into the genome. However, most L1 insertions are truncated at the 5′ end [269], potentially resulting in the loss of promoters, splice acceptors and polyadenylation signals required for mutagenesis [248]. Controlled insertional mutagenesis using L1 derivatives has not yet been reported and *Sleeping Beauty* remains the preferred transposon for cancer gene discovery.

## 3 Cross-species comparative analysis for cancer gene discovery

Important biological sequences, such as gene coding regions and regulatory elements, are conserved in evolution. Cross-species comparative sequence analysis can therefore facilitate the characterisation of known cancer genes. For example, comparison of intronic sequences in human and mouse *BRCA1* led to the identification of two evolutionarily conserved regulatory elements in the second intron that, when mutated, had opposite effects on gene expression [273]. Cross-species comparative analysis also provides an extremely powerful approach for identifying novel genes and gene collaborations involved in cancer formation. Many genes and pathways have been implicated in tumourigenesis, and most human cancers exhibit genomic instability, leading to the acquisition of genetic alterations that drive tumourigenesis but also many passenger mutations that do not contribute to the tumour phenotype. Distinguishing driver and passenger mutations is a major challenge. The underlying molecular mechanisms that govern important biological processes are, however, conserved through evolution and cancer-associated mutation data from other species can therefore be used as a filter for identifying genes that represent strong candidates for a role in human tumourigenesis.

Genome-wide expression data for human tumours can be difficult to interpret, and a number of studies have therefore used cross-species comparative analysis to identify conserved expression signatures that are important in tumourigenesis. Expression profiles of intestinal polyps from patients with a germline mutation in *APC* were compared to those from *Apc*-deficient mice and the conserved signature showed an over-representation of genes involved in cell proliferation and activation of the Wnt/β-catenin signalling pathway [274]. Likewise, comparison of expression profiles for human lung adenocarcinoma and a mouse model of *Kras2*-mediated lung cancer led to the identification of a *KRAS2* expression signature that was not identified by analysing *KRAS2*-mutated human tumours alone [26]. More recently, a mutated *Kras*-specific signature that can be used to classify human and mouse lung tumours on the basis of their *KRAS* mutation status has been identified by comparing *KRAS*-mutated human cancer cells to mouse somatic cells containing knocked-in mutant *Kras* [275].

Mouse prostate cancers induced by human *MYC* have an expression signature that defines a set of “*Myc*-like” human prostate tumours and includes overexpression of the oncogene *Pim1* [236]. Rat prostate tumours also have a similar expression profile to human prostate tumours, and have been used to identify conserved genes that are differentially expressed in both species in response to treatment with the chemopreventive agent Selenium [276]. The mouse is therefore not the only cancer model that has been used for cross-species comparison. The greater the evolutionary distance between the species, the greater the likelihood that conserved changes in gene expression contribute to the cancer phenotype. An expression signature in zebrafish liver tumours is more consistently associated with human liver tumours than with other human tumour types and, since human and zebrafish are distantly related, genes in the conserved signature are strong candidates for a role in cancer development [277].

Another approach for cross-species analysis involves comparing the CGH profiles of human tumours to the CGH profiles of tumours generated from a mouse model of the corresponding human cancer. Such studies take advantage of the conserved synteny between the human and mouse genomes [278]. Comparison of CGH profiles for human neuroblastomas with profiles for tumours and cell lines from a *MYCN* transgenic mouse model of neuroblastoma have shown that many genetic aberrations are conserved between species [279,280]. Likewise, 80% of aberrations detected by array CGH in tumour cells of the mouse model for epithelial ovarian cancer are conserved in human epithelial ovarian cancer [281] and epithelial carcinomas in mice with telomere dysfunction show numerous copy number changes in regions syntenic to those in human cancers [282]. Zender et al. [283] used array CGH to identify regions of copy number change in the tumours of a mouse model for hepatocellular carcinoma. The CGH profiles were compared to array CGH data for human hepatocellular carcinomas to identify minimally conserved amplicons, and genes that showed increased expression in both species were chosen as candidate cancer genes. The authors identified 2 oncogenes, *cIAP1* and *Yap*, that act synergistically in a focal amplicon on mouse chromosome 9qA1, which is syntenic to an 11q22 amplicon in human tumours. Kim et al. [284] used a comparable approach to identify *Nedd9* as a candidate for a role in promoting metastasis of melanomas. A focal amplicon comprising 8 genes, including *Nedd9*, was identified on chromosome 13 in 2 metastatic cell lines derived from a *Ras* mouse model of nonmetastic melanoma. 36% of metastatic melanomas contained a much larger amplicon in a syntenic region on human chromosome 6p25-24, and 35–52% of metastatic melanomas showed significant overexpression of *NEDD9*, with more advanced tumours showing higher levels.

Comparison of human cancers with mouse models of cancer relies on the use of mouse models that accurately recapitulate the human cancer [285]. While *cIAP1* and *Yap* overexpression was found to be important in *p53*^−/−^;*Myc*-induced hepatoblasts in the study by Zender et al. [283], neither gene contributed to tumourigenesis in *p53*^−/−^;*Akt* or *Ras* hepatoblasts. Likewise, *Nedd9* did not contribute to melanoma metastasis in the absence of *Ras* or *Raf* activation [284]. Cross-species comparison of genomic profiles for a particular cancer may therefore require some prior knowledge of the genetic events that drive tumourigenesis in that cancer so that an appropriate mouse model can be generated. However, cross-species analysis can also facilitate the selection of a suitable mouse model. Lee et al. [286] used unsupervised hierarchical clustering of expression data from human and mouse hepatocellular carcinomas to identify the mouse models that provided the best fit for human cancers. Mouse and human tumours that clustered together due to similar expression profiles also shared phenotypic characteristics, such as proliferation rate and prognosis [286]. Most genetically engineered mouse models do not show the high levels of chromosome instability associated with human cancers. Mice that are engineered with telomere dysfunction, or defects in DNA damage checkpoints or DNA repair, may therefore represent better models for comparative oncogenomics [287]. Comparative analysis of copy number alterations in chromosomally unstable murine T-cell lymphomas and human solid tumours identified recurrent aberrations in the mouse that are conserved in human T-ALL but also in other human tumour types [287].

Candidate cancer genes can also be identified by comparing expression and CGH profiles for human tumours with mouse insertional mutagenesis screens. Genes in expression signatures associated with distinct subclasses of human acute myeloid leukaemia were significantly correlated with genes nearest to insertion sites in a Graffi 1.4 MuLV mouse model and with candidate leukaemia genes in BXH2 and AKXD mouse models [288]. There was little overlap between the candidates identified by Graffi 1.4 and BXH2/AKXD, demonstrating that retroviral screens involving multiple models and viruses may be required for a more effective cross-species comparison [195]. Amplified regions in human pancreatic cancer have also been shown to contain more CIS in retrovirus-induced murine lymphomas and leukaemias than expected by chance [289]. As discussed previously, insertional mutagenesis ‘tags’ the mutated gene, therefore facilitating cancer gene identification. In contrast, copy number alterations in human cancer can be very large, encompassing many genes, and no systematic approach currently exists for identifying the critical genes within these regions [290]. Thus comparative analysis of oncogenic insertions in mouse tumours and CGH data for human tumours is potentially a very powerful approach for narrowing down the candidates in regions of copy number change.

## 4 Validating candidate cancer genes

In many cases, identifying candidate cancer genes using the methods described above is the first step towards proving that they are involved in cancer development and further functional validation is usually required.

### 4.1 Validating candidate gain of function mutations

Validating candidate gain of function mutations may be achieved in several ways and the approach applied is largely driven by the tissue or organ system in which the candidate oncogene is being studied. Classically, viruses have been used to overexpress candidate cancer genes either by infecting cells *ex vivo* and then transplanting them back into a host, or by injecting viruses directly into the tissue of interest [291]. The haematopoietic system and the mammary gland are particularly amenable to transplantation, while any organ may be injected with viruses. There are now vast collections of cDNAs in retroviral vectors that provide an ‘off the shelf’ resource for overexpressing and validating candidate cancer genes [292, 293]. Transposons such as *Sleeping Beauty* have also been used to deliver a ‘payload’ containing an oncogene into tissues including the liver [294] and brain [295]. In this context, transposons essentially represent an alternative delivery tool to viruses. Where it is desirable to generate large numbers of animals for study, an alternative strategy has been proposed to generate arrays of transposons carrying oncogenic cDNAs in ES cells and to make lines of mice from which transposons may be mobilized somatically, resulting in expression of oncogenic cDNAs when the transposon lands near a suitable promoter [296]. The premise is that an oncogene requires the right level of expression to participate in transformation. While novel, it is unclear how useful this approach will be since construction of transposon arrays is cumbersome, and it is clear that oncogenic cDNAs present in these arrays are not silent or inert so developmental defects resulting from ectopic expression of candidate oncogenes may occur. An alternative strategy to these overexpression approaches is to knockdown expression of a candidate oncogene, since depletion of a gene that is important in driving oncogenesis may result in decreased growth of the tumour, or of a cell line derived from the tumour in culture. A very powerful, but low-throughput, approach is to knock cDNAs into a defined locus such as the Rosa locus in a Lox–Stop–Lox vector and to express them conditionally after expression of Cre recombinase [297,298]. Elegant methods have also been developed to validate fusion genes using ‘invertor alleles’ [299,300].

### 4.2 Validating candidate loss of function mutations

The most high-throughput approach for validating candidate tumour suppressor genes is to knock them down using shRNAs [301,302]. This approach is largely restricted to tissues in which viral delivery of the shRNA can be achieved, as discussed above. There are, however, constructs available to introduce shRNAs into defined locations in the genome such as the Rosa locus. The success of an shRNA depends on the ability of the transcript to be ‘knocked down’ and on the stability of the protein, so it may not be suitable for all genes. One particularly appealing shRNA-based system incorporates tet regulatable elements so that expression of a gene can be switched off and then on again so that its role in tumour initiation and progression can be studied in detail [297].

Clearly the most powerful approach for validating loss of function mutations is to use conditional loss of function alleles in the mouse. Generating conditional alleles in mice is certainly not a high-throughput strategy, since it takes at least a year to generate an allele and to obtain the mice for study. There are, however, extensive programmes such as the Knockout Mouse Programme (KOMP) and the European Conditional Mutant Mouse (EUCOMM) Programme that are generating impressive collections of conditional alleles in ES cells and mice [303,304].

## 5 Concluding remarks

The study of human and mouse cancers has enabled us to get a window on the genetic complexity of the cancer genome. As we go forward, whole cancer genome sequencing is likely to move to the fore as the primary approach applied to cancer genome analysis. While it is likely that this approach will reveal a number of frequently mutated genes that have been missed by other techniques, intuitively it is likely that many genes will be uncovered that are occasionally mutated. Additionally, it is likely that many frequently rearranged regions containing numerous genes will be identified, further complicating the identification of those mutations that drive the tumorigenic process. Deconvoluting this complexity should be enabled by cross-species cancer gene analysis, which, as described above, has already been shown to be a potent approach for cancer gene identification and validation. The limiting factor of the cross-species cancer gene approach has been generating animal models that faithfully recapitulate the human disease although considerable efforts, such as the Mouse Models of Human Cancer Consortium (MMHCC), are being made to redress this limitation. Clearly, mice, in addition to other animal models, will play a major role in furthering our understanding of the cancer genome. Large-scale oncogenomic approaches that incorporate data from both mouse and human and that apply *in vivo* techniques, such as shRNA knockdown and viral mediated overexpression, to validate candidate cancer genes are likely to become commonplace in the cancer research arena [305].

What is certainly clear is that when we look back on this era in cancer research we will realise how little we understood about the genes and pathways associated with cancer formation and the ingenuity of cancers to evolve and overcome all that we throw at them.

## Figures and Tables

**Table 1:** Overview of genomic technologies for cancer gene discovery.

Cancer gene discovery approach	Resolution	Pros	Cons
Gene resequencing	Nucleotide	Can be an accurate way of finding somatic mutations at the nucleotide level.	PCR-based strategies are not readily scaleable to genome-wide and are expensive.
		With new array-based sequence enrichment technology the entire exome can be profiled.	Array-based sequence enrichment is still developmental and many protocols do not reproducibly capture the exome.
Expression analysis	Transcript	Expression data can be used for diagnostic and prognostic purposes.	Because expression profiling is a quantitative measure of gene activity it reports gene expression changes that are both the cause and the effect of genetic and epigenetic changes at the DNA level which often makes the output of these studies an ‘expression signature’ rather than a cancer gene.
		RNA-Seq based approaches can be used for profiling the transcriptome including expression levels, splicing and fusion gene discovery.	
Comparative Genomic Hybridization (CGH)	Megabase	Large complex rearrangements can be discovered using this technique.	Largely outdated by array-based approaches. Not readily scaleable to high-throughput.
			Reveals large regions of rearrangement which may contain many genes so finding causal rearrangements can be difficult.
Array-based Comparative Genomic Hybridization (aCGH)	100s bp	High resolution. SNP-based platforms can report allele-specific changes.	Stromal contamination and immune cell infiltrates can influence the ability of these platforms to determine the copy number of the cancer.
			Like all genomics platforms array-based CGH reports to copy number profile of a population of cells so tumour heterogeneity can be an issue.
Sequencing entire cancer genomes	Nucleotide	Can report nucleotide level variation, copy number information and can also report neutral changes in the genome such as balanced translocations and inversions.	Extremely expensive. Not clear how to computationally resolve highly rearranged regions.
Epigenetic profiling	Nucleotide	Can detect epigenetically silenced genes that would be missed by other approaches.	It has proved difficult to develop technology to scale to genome-wide epigenetic profiling at the nucleotide level.
